# Life-Threatening Postpneumonectomy Syndrome Complicated with Right Aortic Arch after Left Pneumonectomy

**DOI:** 10.1155/2015/768067

**Published:** 2015-05-28

**Authors:** Takahiro Karasaki, Makoto Tanaka

**Affiliations:** Department of Thoracic Surgery, JR Tokyo General Hospital, 2-1-3 Yoyogi, Shibuya-ku, Tokyo 151-8528, Japan

## Abstract

A 54-year-old man with right aortic arch underwent left lower lobectomy and lingular segmentectomy, followed by complete pneumonectomy, for refractory nontuberculous mycobacterial infection. Three months after the pneumonectomy, he developed acute respiratory distress. Computed tomography showed an excessive mediastinal shift with an extremely narrowed bronchus intermedius and right lower bronchus compressed between the right pulmonary artery and the right descending aorta. Soon after the nearly obstructed bronchus intermedius was observed by bronchoscopy, he began to exhibit frequent hypoxic attacks, perhaps due to mucosal edema. Emergent surgical repositioning of the mediastinum and decompression of the bronchus was indicated. After complete adhesiolysis of the left thoracic cavity was performed, to maintain the proper mediastinal position, considering the emergent setting, an open wound thoracostomy was created and piles of gauze were inserted, mildly compressing the heart and the mediastinum to the right side. Thoracoplasty was performed three months later, and he was eventually discharged without any dressings needed. Mediastinal repositioning under thoracostomy should be avoided in elective cases because of its extremely high invasiveness. However, in the case of life-threatening postpneumonectomy syndrome in an emergent setting, mediastinal repositioning under thoracostomy may be an option to save life, which every thoracic surgeon could attempt.

## 1. Introduction

Postpneumonectomy syndrome (PPS) is a rare complication that occurs in 0.16% to 2% of cases after pneumonectomy [1, 2]. It is characterized by an excessive mediastinal shift resulting in bronchial compression and obstruction. Reported symptoms are stridor, dyspnea, and recurrent airway infections. Diagnosis is based on computed tomography (CT) and bronchoscopy. After right pneumonectomy, the left main bronchus is compressed between the aortic arch and the pulmonary artery. After left pneumonectomy, the elongated right main bronchus is compressed between the pulmonary artery and the thoracic spine, although there are fewer symptomatic cases than after right pneumonectomy. On the other hand, in a patient with right aortic arch, which is a rare congenital malformation that reportedly is found in approximately 0.1% of the population, the right main or intermediate bronchus is subject to compression between the pulmonary artery and the right descending aorta after left pneumonectomy [3, 4]. We report a case with right aortic arch who presented with life-threatening PPS after left pneumonectomy for refractory nontuberculous mycobacterial infection.

## 2. Case Presentation 

**Figure 1:**
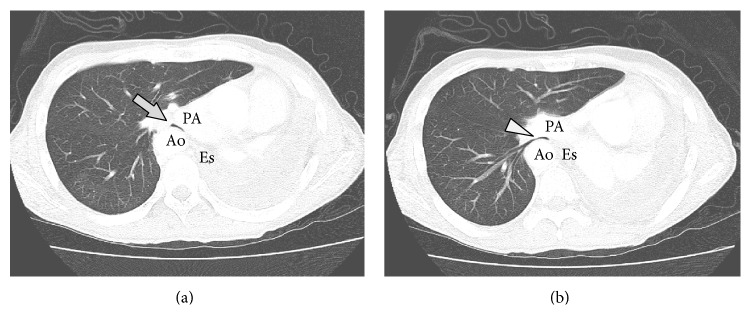
Computed tomography taken after the development of postpneumonectomy syndrome. Extremely narrowed bronchus intermedius ((a) arrow) and right lower bronchus ((b) arrowhead) are shown, compressed between the right pulmonary artery and the right descending aorta. Ao, right descending aorta; PA, right pulmonary artery; Es, esophagus.

**Figure 2:**
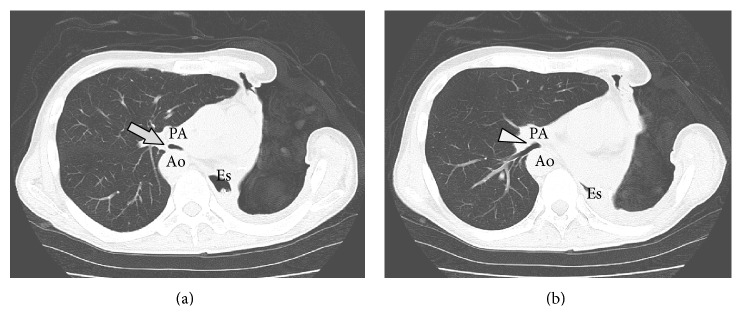
Computed tomography taken two weeks after mediastinal repositioning. Complete adhesiolysis of the left thoracic cavity was performed and a thoracostoma was created. Piles of gauze were inserted to maintain the proper mediastinum position. The compression of the bronchus was released, and the lumens of the bronchus intermedius ((a) arrow) and right lower bronchus ((b) arrowhead) were sufficiently patent. Ao, right descending aorta; PA, right pulmonary artery; Es, esophagus.

**Figure 3:**
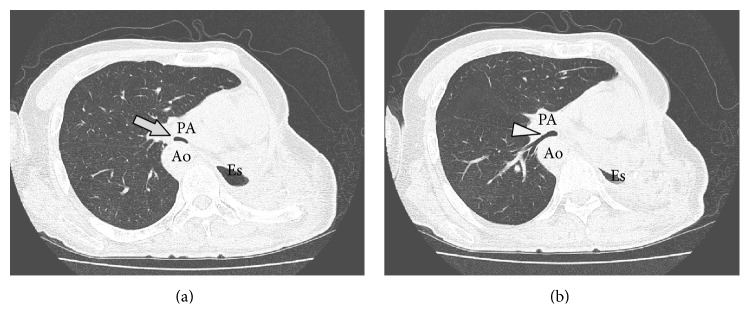
Computed tomography taken two months after the thoracoplasty. The bronchus intermedius ((a) arrow) and right lower bronchus ((b) arrowhead) are patent, and there is no relapse of postpneumonectomy syndrome. Ao, right descending aorta; PA, right pulmonary artery; Es, esophagus.

A 54-year-old man with right aortic arch and Kommerell's diverticulum presented with refractory hemoptysis due to a rapidly growing mycobacterium avium complex (MAC) infection, which could not be controlled by bronchial artery embolization. Left lower lobectomy and lingular segmentectomy were performed. Although antimycobacterial agents were continued, MAC infection relapsed in the left upper segment. Eight months after the initial surgery, complete pneumonectomy was performed. The lung was strongly adherent to pericardium and diaphragm. The adherent lung was slightly injured after the adhesiolysis, and the chest cavity was minimally contaminated. The wound was closed after thorough irrigation of the chest cavity. The postoperative course was uneventful and the patient was discharged on postoperative day 14. Three months after the pneumonectomy, he developed shortness of breath. He had neither a history of asthma nor congenital heart disease. The symptom worsened over the course of several days, and he was transferred to the emergency department with acute respiratory distress. He was intubated with a single-lumen orotracheal tube, and positive pressure ventilation was initiated. CT scan showed a narrowed bronchus intermedius and the right lower bronchus compressed between the right pulmonary artery and the right descending aorta, although the airway was barely patent (Figure 1). Bronchoscopy revealed an almost completely obstructed bronchus intermedius. The patient became hypoxic soon after bronchoscopy, perhaps due to mucosal edema. Ventilation was maintained by systemic steroid and transbronchial administration of diluted adrenaline, but the hypoxic attacks frequently relapsed. Before the airway was completely obstructed, percutaneous cardiopulmonary support was initiated via a femoral artery and vein. The endotracheal tube was then replaced by a double-lumen endobronchial tube under bronchoscopy. The tip of the tube was placed into the bronchus intermedius, and the second lumen orifice was open to ventilate the right upper lobe. One of the treatment options was temporary placement of a bronchial stent, although the feasibility and efficacy were uncertain. Permanent placement of a bronchial stent was also not indicated due to the risk of aortobronchial fistula. Thus surgical repositioning of the mediastinum was indicated. The left thoracic cavity was reopened, and complete adhesiolysis was performed. To maintain the proper mediastinal position, considering the emergent setting, an open-window thoracostomy was created with resection of the second to ninth ribs, and piles of gauze were inserted, mildly compressing the heart and the mediastinum to the right side. The inserted gauze and dressings were exchanged every day. The patient was extubated on postoperative day 5. CT scan showed a repositioned mediastinum and sufficiently patent airways (Figure 2). Three months after repositioning of the mediastinum, the thoracostoma was closed with a latissimus dorsi muscle flap covering the heart. Eventually, the patient was discharged without any dressings needed. Two months after thoracoplasty, no relapse of PPS has been seen so far (Figure 3).

## 3. Discussion 

From 1979 to 2014, a total of 102 adult cases of PPS have been reported in the English literature, 72 after right pneumonectomy and 30 after left pneumonectomy [1, 5–11]. Among the 30 cases of PPS after left pneumonectomy, 6 (20%) had right aortic arch [1]. Although the exact incidence of PPS after left pneumonectomy with right aortic arch cannot be estimated, right aortic arch undoubtedly is associated with a high risk of PPS after left pneumonectomy.

The most commonly used treatment in PPS is surgical repositioning of the mediastinum with thorough adhesiolysis followed by the implantation of fixed-volume or expandable prostheses [1, 5]. The use of saline-filled breast implants or silicone prosthesis has been reported elsewhere, although luxation, malposition, herniation, and leakage after prosthesis implantation have been reported [1, 5]. The insertion of a tracheobronchial stent may be an option, although the long-term results are of concern, considering the risk of stent migration and fistulation with the pulmonary artery or descending aorta [5]. Other surgical techniques described in the literature include pericardial fixation at the sternum, anterior resection of a vertebral body, and complex reconstruction such as aortic division and bypass or tracheobronchial reconstructions. The efficacy and necessity of these procedures are unclear [1]. A less-invasive method is preferable, although very few surgeons have encountered this rare disease. In the present case, we decided to perform mediastinal repositioning with thoracostomy for several reasons. There was a high risk of complete airway obstruction even with slight dislocation of the endobronchial tube, which necessitated an emergent operation. Considering the risk of empyema, implantation of prosthesis seemed unsuitable for the present case.

Mediastinal repositioning with thoracostomy is unquestionably much more invasive and mandates an impaired quality of life compared to prosthesis implantation with a one-stage open or thoracoscopic approach. The present method should also not be recommended in elective cases with mild symptoms. However, it may be helpful in cases of severe PPS in an emergent setting. Mediastinal repositioning under thoracostomy may be a life-saving treatment option which every thoracic surgeon could attempt without the use of any unfamiliar devices.

## References

[1] Soll C., Hahnloser D., Frauenfelder T., Russi E. W., Weder W., Kestenholz P. B. (2009). The postpneumonectomy syndrome: clinical presentation and treatment. *European Journal of Cardio-Thoracic Surgery*.

[2] Jansen J. P., De la Riviere A. B., Alting M. P. C., Westermann C. J. J., Bergstein P. G. M., Duurkens V. A. M. (1992). Postpneumonectomy syndrome in adulthood; surgical correction using an expandable prosthesis. *Chest*.

[3] Hastreiter A. R., D'Cruz I. A., Cantez T., Namin E. P., Licata R. (1966). Right-sided aorta. I. Occurrence of right aortic arch in various types of congenital heart disease. II. Right aortic arch, right descending aorta, and associated anomalies. *British Heart Journal*.

[4] Grillo H. C., Shepard J.-A. O., Mathisen D. J., Kanarek D. J. (1992). Postpneumonectomy syndrome: diagnosis, management, and results. *Annals of Thoracic Surgery*.

[5] Shen K. R., Wain J. C., Wright C. D., Grillo H. C., Mathisen D. J. (2008). Postpneumonectomy syndrome: surgical management and long-term results. *The Journal of Thoracic and Cardiovascular Surgery*.

[6] Kalluri M., Sahn S. A., Highland K. B. (2008). Chronic dyspnea and severe obstruction after pneumonectomy: postpneumonectomy syndrome. *The American Journal of the Medical Sciences*.

[7] Ng T., Ryder B. A., Maziak D. E., Shamji F. M. (2009). Thoracoscopic approach for the treatment of postpneumonectomy syndrome. *Annals of Thoracic Surgery*.

[8] Partington S. L., Graham A., Weeks S. G. (2010). Pulmonary vein stenosis following left pneumonectomy: a variant contributor to postpneumonectomy syndrome. *Chest*.

[9] Bastin A. J., Karunanantham J., Lim E. (2010). Breathlessness after pneumonectomy: consider postpneumonectomy syndrome. *Clinical Lung Cancer*.

[10] McRae M. C., Detterbeck F. C., Narayan D. (2011). Correction of postpneumonectomy syndrome using a custom implant. *BMJ Case Reports*.

[11] Matheson J. A., Lohn J. W., Antippa P. N., MacGill K. (2014). Intrathoracic tissue expanders for postpneumonectomy syndrome. *Journal of Plastic, Reconstructive & Aesthetic Surgery*.

